# Supplementary material for: In-situ observation of trapped carriers in organic metal halide perovskite films with ultra-fast temporal and ultra-high energetic resolutions
Source: Nat Commun. 2021 Mar 12;12:1636. doi: 10.1038/s41467-021-21946-2 (PMC7954808; doi:10.1038/s41467-021-21946-2)
Supplement: Supplementary file 1 — Supplementary Information [file 41467_2021_21946_MOESM1_ESM.pdf]

Supplementary Information

In-situ Observation of Trapped Carriers in Organic Metal Halide Perovskite Films with Ultra-fast Temporal and Ultra-high Energetic Resolutions

Kanishka Kobbekaduwa[1†], Shreetu Shrestha[2†], Pan Adhikari[1], Exian Liu[1], Lawrence Coleman[1], Jianbing Zhang[3], Ying Shi[4], Yuanyuan Zhou[5], Yehonadav Bekenstein[6], Feng Yan[7], Apparao M. Rao[1], Hsinhan Tsai[2], Matthew C. Beard[8], Wanyi Nie*[2] and Jianbo Gao*[1]

[1] Department of Physics and Astronomy, Ultrafast Photophysics of Quantum Devices Laboratory, Clemson University, Clemson, SC 29634, United States

[2] Center for Integrated Nanotechnology, Los Alamos National Laboratory, Los Alamos, NM 87545, United States

[3] School of Optical and Electronic Information, Huazhong University of Science and Technology, Wuhan 430074, P. R. China

[4] Institute of Atomic and Molecular Physics, Jilin Provincial Key Laboratory of Applied Atomic and Molecular Spectroscopy, Jilin University, Changchun, 130012, P. R. China

[5] Department of Physics, Hong Kong Baptist University, Kowloon Tong, Hong Kong, P. R. China

[6] Department of Materials Science and Engineering, Technion, Haifa 32000, Israel

[7] Department of Metallurgical and Materials Engineering, The University of Alabama, Tuscaloosa, AL 35487, United States

[8] National Renewable Energy Laboratory, Golden, CO 80401, United States

Corresponding author emails: wanyi@lanl.gov; jianbogao.nano@gmail.com

1. **Supplementary Figures……………………………………………………….……………..3**

Supplementary Fig. 1-8

2. **Supplementary Methods…………………………………………………………………...8**

Supplementary Fig. 9

3. **Supplementary Note 1…………………………...…………………………………………11**

4. **Supplementary Note 2……………………………………...…………………………….12**

5. **Supplementary Note 3…………………………...…………………………………………13**

Supplementary Fig. 10-12

6. **Supplementary Table of the trap properties….…………………………………………..20**

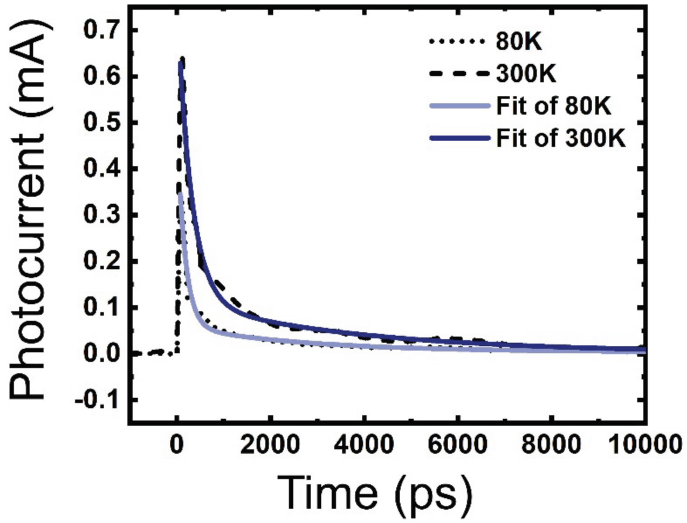

Supplementary Fig. 1: **Ultrafast photocurrent in a longer time window.** The bi-exponential fitting of the ultrafast photocurrent decay at two different temperatures (300K and 80K) gives a lifetime of ~1 ns for the $MAPbI_3$ thin films. This value is for photocurrent lifetime in-situ devices under an external electric field and is different from 'traditional' $MAPbI_3$ lifetimes obtained from steady state photoluminescence measurements which represent ex-situ materials.

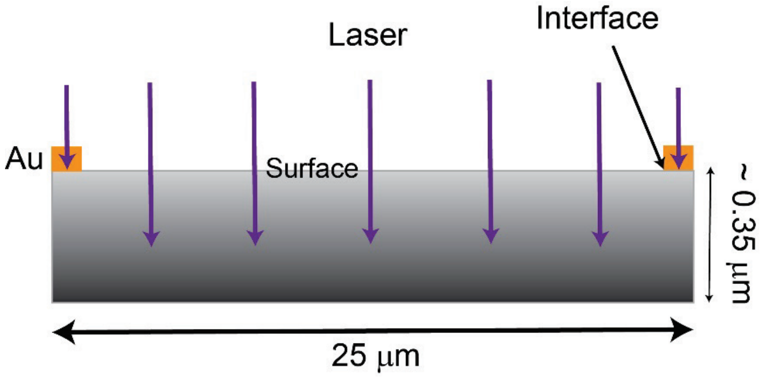

Supplementary Fig. 2: **Device cross-section.** Photon absorption depth is ~200 nm whilst the film thickness is ~350 nm. Hence, majority of the charge carriers excited by 3.1 eV light are from the bulk thus, interface and surface effects on photocurrent measurements are negligible.

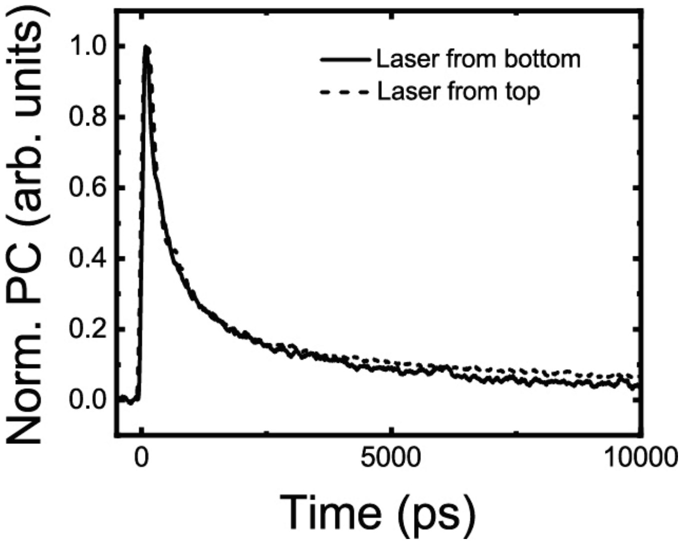

Supplementary Fig. 3: **Ultrafast photocurrent taken from both sides of the films**. Comparison of ultrafast photocurrent with laser illumination from the top surface of device (dotted line) and through the quartz substrate from the bottom. The applied electric field is 10^4 V/cm, laser flux of 0.4 μJ/cm^2 and temperature is 300 K.

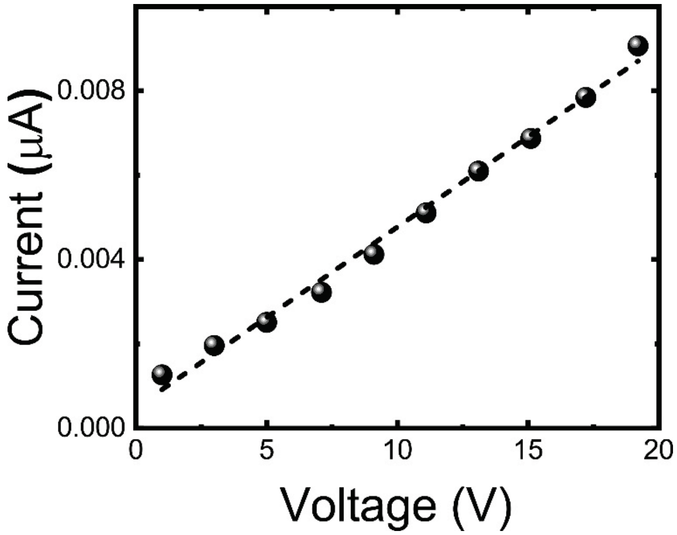

Supplementary Fig. 4: **Dark IV curve.** The dark IV curve at 300 K measured across the gap. At low voltages, the contact shows an Ohmic behavior. The range considered is only 0-20 V as at higher voltage values, ion migration will cause the curve to deviate from the ohmic regime.

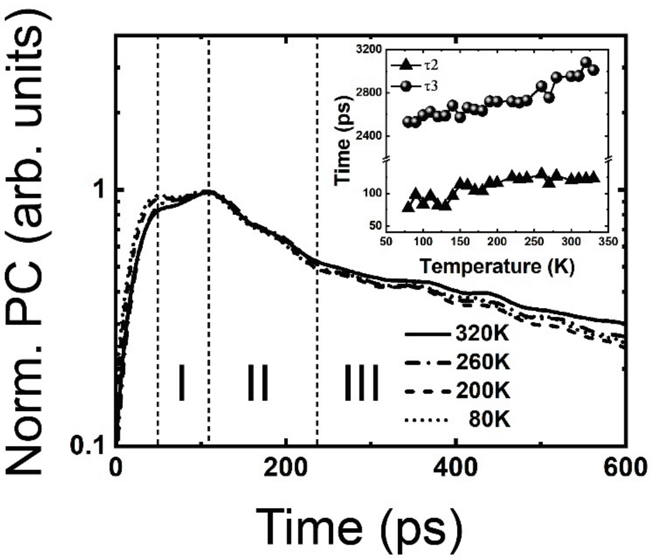

Supplementary Fig. 5: **Temperature dependence of ultrafast photocurrent.** The normalized ultrafast photocurrent decay for a second device at various temperatures to demonstrate that the transport properties are the same for all devices studied. The inset is decay constants dependence with temperature of region II and region III.

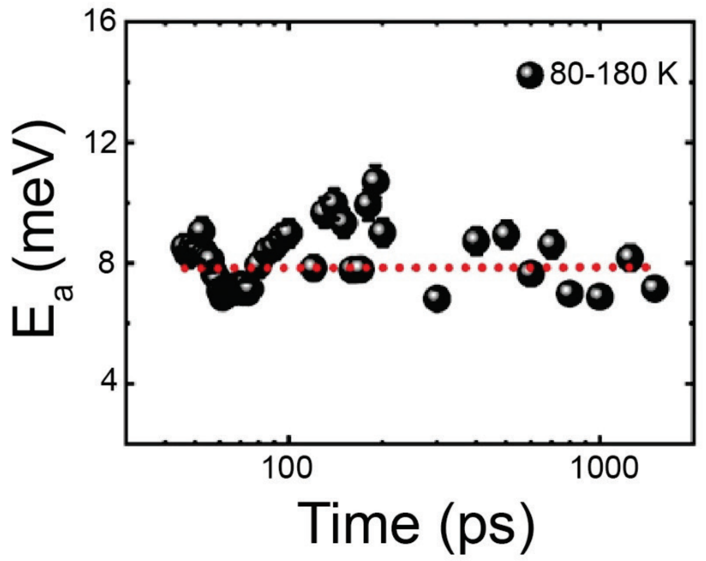

Supplementary Fig. 6: **Activation energy variation in the 80-180K temperature range.** The dotted line represents the linear fitting for the variation from 25 ─ 2000 ps**.**

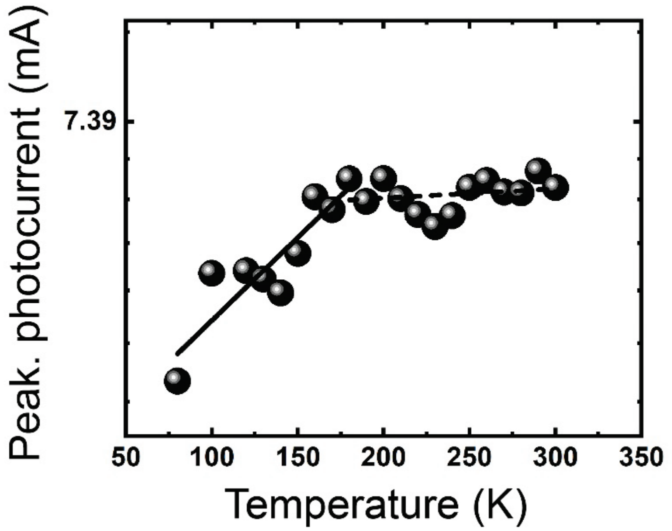

Supplementary Fig. 7: **Photocurrent peak dependence with temperature.** The shown peak dependence indicates the slightly increasing thermal activation behavior.

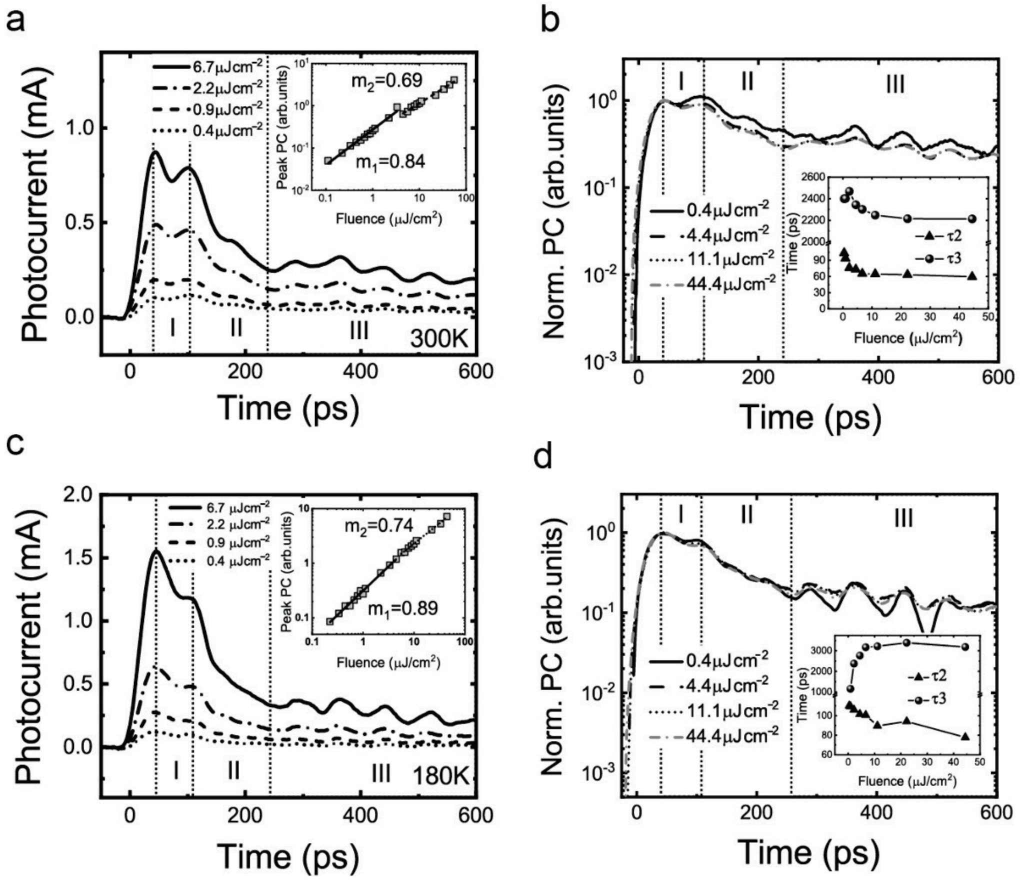

Supplementary Fig. 8: **Ultrafast photocurrent dependence on laser intensity.** Ultrafast photocurrent at 300 K (**a)** and 80 K (**c**). The photocurrent peak dependence with the excitation intensities is shown in the inset along with the slope value. The normalized photocurrent with rates of decay (insets) in regions II and III for 300 K **(b)** and 80 K **(d)** were found via bi-exponential fitting.

Supplementary Methods

Materials

Lead iodide (PbI_2), Methylammonium iodide (MAI), Dimethylformamide (DMF) and Dimethyl sulfoxide (DMSO) purchased from Sigma-Aldrich and used without further purification.

Perovskite film

The initial precursor is synthesized by dissolving 360 mg PbI_2 and an equimolar ratio of MAI in 0.5 ml of DMF and 0.5 ml DMSO at 70 °C (343 K) with constant stirring for 24 hours. To reduce contaminants and unwanted reactions, deposition cannot be done under air. Hence, the substrates are transferred into an argon filled glove box and heated at 170 °C. Finally, Perovskite layers are deposited via the hot-casting method, where the precursor is briefly heated at 70 °C and spin coated onto the preheated substrate at 5000 rpm for 20 s. Supplementary Fig. 9 shows the morphology images of the film at different stages of synthesis[1].

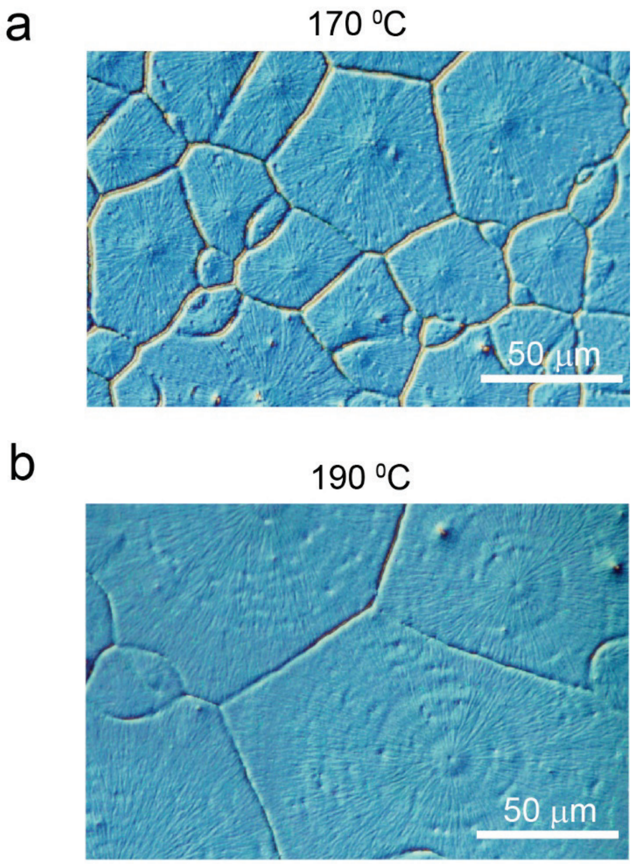

Supplementary Fig. 9**: Surface morphology images:** The hot casted MA perovskite films at **a.** 170^{0}C and **b.** 190^{0}C during the synthesis process.

Experimental setup

The high-speed photoconductive devices are fabricated by the integration of a coplanar microstrip transmission line waveguide and $MAPbI_3$ thin film photoconductors. After the synthesis of $MAPbI_3$ using the procedure described above, a layer of material is deposited on a glass substrate by spin coating to form a 350 nm thin film. The subsequent thin film is then integrated with coplanar gold (Au) transmission lines. The transmission lines are separated by a 25 μm gap by thermal evaporation through a shadow mask. The transmission line structure greatly reduces device RC time constant (τ_{RC}) by reducing device area and the electrode spacing since $C = \frac{A\varepsilon}{d}$,

where A, d, and ε is the area of the active device (< 5×10^{-5} cm^2), d the electrode spacing (25 μm), and permittivity of quartz ($4.5\varepsilon_0$, $\varepsilon_0 \approx 9\times10^{-12}$ $C^2N^{-1}m^{-2}$)[2], respectively. The calculated C value is, ~0.4 pF.

Photocurrent measurement is done by integrating the above devices within a custom-built sample holder which is put under a vacuum environment via a cryostat. The cryostat provides a sealed environment; in which, the experimental temperature can be varied while keeping the system under a vacuum. This setup prevents ionization reactions with air and electrical arcing within the device at high electric fields. The required electric field is generated via a source meter, and the photocurrent is collected by a 40 GHz sampling oscilloscope. A 400 nm (3.1 eV) ultrafast laser with a 130 fs pulse illuminates the complete device area and excites carriers up to ~200 nm deep in the ~0.35 μm (350 nm) thin film as shown in Fig. 1. An ultrafast response time of 25 ps was achieved. The limiting factors for this fast time resolution were the bandwidths of photoconductive devices, the 40 GHz sampling oscilloscope, cables, and connectors.

Supplementary Note 1

Carrier mobility calculations

The mobility calculated in this work is essentially a combination of both electrons (μ_e) and holes (μ_h) since we cannot identify each separately using our technique. Thus, the effective mobility (μ_{eff}=μ_e+μ_h) of the perovskite film can be calculated by[3,4],

$$\mathbf{I_P} = e\eta\mu_{eff}N\frac{E}{L} \quad (1)$$

where $\mathbf{I_P}$ is the photocurrent peak, e is the electron charge, η is the quantum yield, μ_P is the mobility, N is the total number of photons, E is the electric field, and L is the width of the gap between the electrodes. We can write the electric field as, $\mathbf{E} = \frac{V}{L}$, where V is the bias voltage. Thus, Supplementary Equation (1) becomes,

$$\mathbf{I_P} = e\eta\boldsymbol{\mu}_{eff}N\frac{V}{L^2} \quad (2)$$

Although N is the total number of photons, to calculate the actual mobility we require the effective number of photons N_{eff} (*i.e.* the number of photons that incident on the device area).

If I is the measured intensity of our laser and $\boldsymbol{\hbar\omega}$ is the energy per photon, then the total number of photons generated per second is, $n = \frac{I}{\hbar\omega}$. In term of experimental conditions, the laser wavelength is 400 nm (3.1 eV), the laser intensity I is 2 µW, the laser beam size is 150 µm x 6 mm, and the active device area is 25 µm x 2 mm. Hence, we reach the effective intensity: $I_{Eff} = \frac{2\times10^{-6}\times25\times2}{150\times6} \approx 111$ nW.

For 2 µW we have $n = 4\times10^{12}$ s^{-1}. Then the total number of photons per one pulse is,

$$N = \frac{n}{f} \quad (3)$$

where f is the frequency of the laser. Our laser operated at 1kHz; therefore, we have $N = 4\times10^9$.

The effective number of photons is then: $N_{\text{Eff}} = \frac{4\times10^9}{18} \approx 2.22\times10^8$.

If we assume η, or quantum yield, is equal to 100% (or 1), the width of the gap $L = 25$ µm, $V = 60$ V, $e = 1.6\times10^{-19}$ C, and the resultant peak current is, I_P=1.1 mA. Mobility is given by,

$$\mu_{\text{eff}} = \frac{I_P L^2}{e\eta N_{\text{Eff}} V} \tag{4}$$

$$\mu_{\text{eff}} = \frac{1.1\times10^{-3}\times(25\times10^{-4})^2}{1.6\times10^{-19}\times1\times2.22\times10^8\times60} \approx 3.6\ \text{cm}^2\text{V}^{-1}\text{s}^{-1}$$

Note: the mobility calculation is the lower estimated value because we assume the quantum yield is 100 %.

Supplementary Note 2

External quantum efficiency (EQE) calculations

The external quantum efficiency(φ) is the percentage of excited electrons to the number of photons and is given by,

$$\varphi = \frac{N_e}{N} \tag{5}$$

where N_e is the total number of excited electrons and N is the total number of photons.

To find the total number of electrons, we require the total charge of all the excited electrons Q_e that is calculated from the area of the photocurrent vs time curve. As the data is initially fitted with a 2 exponential decay fit, the total charge is then given by[5],

$$Q_e = \int_{t1}^{t2} \left(A_1\, e^{-\frac{t}{\tau 1}} + A_2 e^{-\frac{t}{\tau 2}}\right) \text{dt} \tag{6}$$

We know, $N_e = \frac{Q_e}{e}$. From the plot we find that $Q_e \approx 8\times10^{-13}$ C at room temperature (300 K) when $V = 60$ V and $I = 2$ μW. Thus, the equivalent number of charge carriers is $N_e = 5\times10^6$. The carrier density is given by, $C_D = \frac{N_e}{Lwd}$ where w (2 mm) is the width of the electrode and d (350 nm) is the thickness of the thin film. Thus, we have $C_D = 2.8\times10^{14}\text{cm}^{-3}$.

Circling back to Supplementary Equation (5), we find the quantum efficiency at $V = 60$ V and $I = 2$μW will be $\varphi \approx 2.25\%$.

Supplementary Note 3

Trap property calculations

To define trapping dynamics, we need to consider several trap related properties.

1. Trap depth
2. Trap density
3. De-trapping rate
4. Capture cross-section
5. Trapping rate

Trap depth (E_t)

The trap depth is calculated directly from the temperature dependent ultrafast photocurrent. As mentioned in the main text, the temperature(T) dependent photocurrent can be described by an Arrhenius equation,

$$\mathbf{I}(T) = I_0 e^{\left(-\frac{E_t(t)}{k_B T}\right)} \quad (7)$$

where E_t is the activation energy, k_B is the Boltzmann constant and I_0 is current at $E_t = 0$. Thus, from the slopes (slope×k_B) of ln(**I**(*t*)) vs 1/*T* we can calculate activation energies in each region (see Fig. 2b, 2c and 2d). These activation energies are equal to the trap depths.

Case study:

The ln (*I*) *vs.* 1/*T* plot for region I is shown in Fig. 2b. The activation energy at 57.5 ps in the temperature range 180 K-320 K is,

$$E_t = \frac{13.56\times1.38\times10^{-23}}{1.6\times10^{-19}} \approx 1.17 \text{ meV}$$

Trap density (N_T)

The trap density can be found via integration of the photocurrent curve, as in Supplementary Equation (6). Demonstrated in the inset of laser fluence dependence at 300 K (Supplementary Fig. 8a), the change of the peak photocurrent with respect to laser fluence from linear to sub linear dependence suggests complete trap filling at around 6.7 μJcm^{-2}. Additionally, if we accept the premise that all carriers that fall into traps are de-trapped and are collected by the measurement system, the total carrier density calculated from the photocurrent at 6.7 μJcm^{-2} should be equal to the total trap density N_T. Thus, for regions I, II, and III, the trap densities N_{T1}, N_{T2}, and N_{T3} can be found by integration of the total curve from 25 ps, 100 ps, and 220 ps respectively.

Case study:

Trap density in region I: $N_T = \frac{\int_{25}^{100} I(t)dt}{eAL} \approx \frac{311\times10^{-15}}{1.6\times10^{-19}\times9\times10^{-6}\times25\times10^{-4}} \approx 3\times10^{15} \text{ cm}^{-3}$

De-trapping rate (k_{th})

De-trapping is essentially carrier emission from trapped states. We introduce that carrier emission is due to a *phonon-assisted tunneling* mechanism (PAT), attributed to the initial de-trapping

process in the ultra-shallow, localized states. The PAT model is validated by the initial decay/rise process in the photocurrent spectra and the decay rate fitting in Fig 2. According to the phonon assisted tunneling mechanism, the emission rate k*(E)* can be written as[6],

$$k(E) = k(0)e^{\left(\frac{E^2}{E_C^2}\right)} \quad (8)$$

where E is the electric field, and E_C is a characteristic field equal to $\sqrt{\frac{3m_e^*\hbar}{e^2\tau^3}}$ (m_e^* is the effective mass and k(0) is the zero-field emission rate. For $MAPbI_3$ the effective mass $m_e^* \approx 0.23m_0$ ($m_0 = 9.11\times10^{-31}$ kg)[7], ħ is the Planck's constant and τ is the tunneling time which is dependent on temperature).

From Supplementary Equation (8) we see that the emission rate increases with the increase in electric field. This leads to an increase in de-trapping which reduces the decay rate, *R(E)* (as seen in the initial increase in the decay time in the inset of Fig. 4b). Thus, k(E) ∝ $\frac{1}{R(E)}$ which leads to

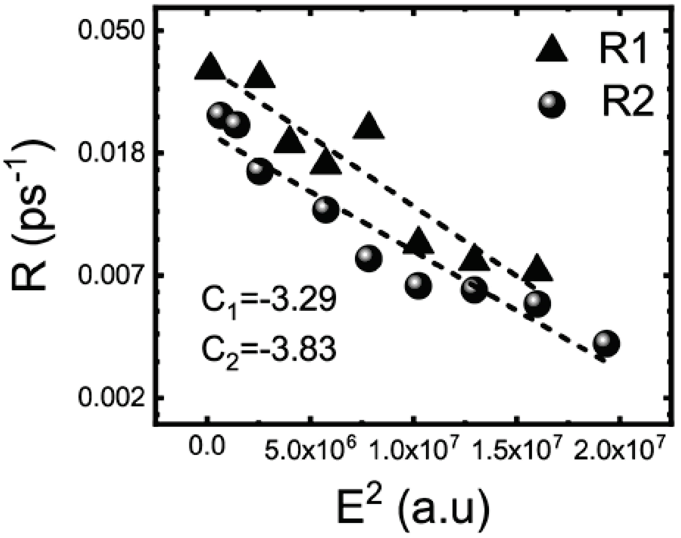

Supplementary Fig. 10: **Zero field emission rate at 300 K according to the phonon-assisted-tunneling model.** Depending on each region we have R1, R2 which correspond to region I, and II respectively.

$\mathrm{R}(E) = \mathrm{R}(0)\mathrm{e}^{\left(-\frac{E^2}{E_C^2}\right)}$. Assuming at zero E-field the emission rate is equal to the decay rate, the de-trapping rate is found by intercept fitting (ln R(0)) of ln R vs E^2 (k_{th}=$R(0)$) .

Case study:

For a constant temperature (300 K) according to phonon assisted tunneling model the intercept from R1 fitting as seen in Supplementary Fig. 10 for region I is -3.29. Thus, the de trapping rate is,

$$k_{\mathrm{th}} \approx \mathrm{R}(0) = \mathrm{e}^{C_1} = 3.7\times10^{10}\ \mathrm{s}^{-1}$$

We can also find the corresponding tunneling time from the slope of the fitting.

$$\mathrm{slope} = \frac{1}{\mathbf{F}_C^2} = \frac{e^2\tau^3}{3m^*\hbar}$$

$$\tau = \left(\frac{3m_e^*\hbar\times slope}{e^2}\right)^{\frac{1}{3}} = \left(\frac{3\times0.23\times9.11\times10^{-31}\times1.05\times10^{-34}\times9.7\times10^{-12}}{(1.6\times10^{-19})^2}\right)^{\frac{1}{3}}$$

$$\tau \approx 0.3\ \mathrm{ps}$$

Capture cross-section (σ_c)

As mentioned above, the thermal de-trapping rate (k_{th}) is equal to the zero-field emission rate *k(0).* Because the thermal de-trapping rate is parallel to the photocurrent decay, it follows an Arrhenius relation similar to Supplementary Equation (7). Hence, we can write k_{th} with respect to temperature in an exponential term,

$$k_{\mathrm{th}} = \mathrm{p}(0)\mathrm{e}^{\left(-\frac{E_{t(t)}}{k_B T}\right)} \quad (9)$$

Here, p(0) is a material specific constant that describes trapping characteristics and is given by $\mathrm{p}(0) = N_c \upsilon_{th} \sigma_c$. N_c is the effective density of states given by $N_c = 2\left(\frac{2\pi m_e^* k_B T}{h^2}\right)^{\frac{3}{2}}$($h$ is the Planck

constant), v_{th} is the thermal velocity of the carriers given by $v_{th}=\sqrt{\frac{3k_BT}{m_e^*}}$, and σ_c is the capture cross section, allowing the capture cross-section to be written as,

$$\sigma_c = \frac{k_{th}}{N_c \upsilon_{th}} e^{\left(\frac{E_t}{k_B T}\right)} \tag{10}$$

Case study:

Then, we have

$$N_c = 2\left(\frac{2\times\pi\times 0.23\times 9.11\times 10^{-31}\times 1.38\times 10^{-23}\times 300}{(6.62\times 10^{-34})^2}\right)^{\frac{3}{2}} \approx 2.8\times 10^{18}\ \text{cm}^{-3}$$

$$v_{th} = \left(\frac{3\times 1.38\times 10^{-23}\times 300}{0.23\times 9.11\times 10^{-31}}\right)^{\frac{1}{2}} \approx 1.3\times 10^{8}\ \text{cms}^{-1}$$

Hence for $k_{th} = 3.7\text{x}10^{10}\ \text{s}^{-1}$ the capture cross-section is,

$$\sigma_C = \frac{k_{th} e^{\left(\frac{E_t}{k_B T}\right)}}{N_C v_{th}} = \frac{3.7\times 10^{10}\times 0.94}{2.8\times 10^{24}\times 1.3\times 10^{6}} \approx 1\times 10^{-16}\ \text{cm}^{-2}$$

Trapping rate (k_{tr})

To calculate the carrier trapping rate, we primarily consider the temperature dependence of trapping. As we see in Fig. 2, in the PAT model the trapping – de-trapping process is directly influenced by temperature. We can write this behavior as,

$$k'(T) = k'(0) e^{\left(-\frac{\gamma}{T}\right)} \tag{11}$$

Where, $k'(0)$ is the zero temperature constant and $\gamma = E_t - \beta E^n$. From the inset of Fig. 2a, we see that the decay time increases (decrease of decay rate) with temperature. This implies $k'(T) \propto \frac{1}{R'(T)}$, giving us $R'(T) = R'(0)\exp^{\left(-\frac{T}{\gamma}\right)}$. Assuming at zero temperature only trapping occurs in the device, the trapping rate can be inferred from R'(0) from the intercept ln R'(0) of ln R' vs T (k_{tr}=R'(0)).

Case study:

In region I the trapping rate can be found from the intercept of R1 in Supplementary Fig. 11. The trapping rate is,

$$k_{\mathrm{tr}} \approx \mathrm{R}^{'}(0) = \mathrm{e}^{C_1} \approx 1.1\times10^{11}\mathrm{s}^{-1}$$

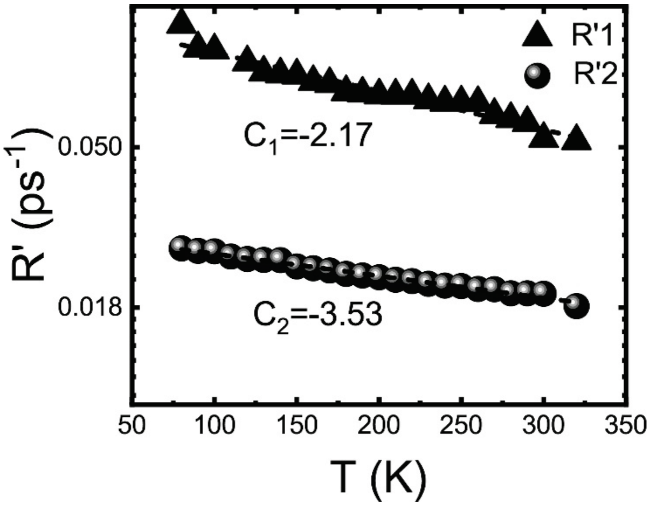

Supplementary Fig. 11: **Zero temperature decay rate at constant electric field.** Depending on each region we have R'1, R'2 which correspond to region I, and II respectively.

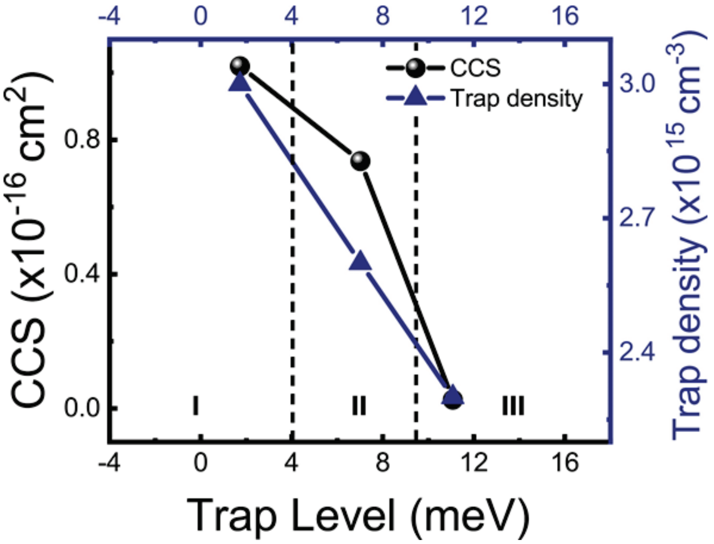

Supplementary Fig. 12: **Trap state distribution.** Capture cross sections and trap densities mapped with respect to the trap levels. The illustrations show how the trap size and density changes from region I to III.

Supplementary table of the trap properties

Supplementary Table 1: **Comparison between trap parameters at 3 different regions.** The parameters are calculated at 300K.

Region	Trap level (E_a) (meV)	Trap density (cm^{-3})	Capture cross section (cm^2)	Trapping rate (s^{-1})	De-trapping rate (s^{-1})	Tunneling time (ps)
I	1.72	3×10^{15}	1.02×10^{-16}	1.14×10^{11}	3.7×10^{10}	0.29
	2.02	3×10^{15}	1.03×10^{-16}	1.14×10^{11}	3.7×10^{10}	0.29
	2.23	3×10^{15}	1.04×10^{-16}	1.14×10^{11}	3.7×10^{10}	0.29
II	4.51	2.6×10^{15}	6.68×10^{-17}	2.94×10^{10}	2.17×10^{10}	0.31
	5.82	2.6×10^{15}	7.03×10^{-17}	2.94×10^{10}	2.17×10^{10}	0.31
	7.02	2.6×10^{15}	7.37×10^{-17}	2.94×10^{10}	2.17×10^{10}	0.31
	8.05	2.6×10^{15}	7.66×10^{-17}	2.94×10^{10}	2.17×10^{10}	0.31
III	10.33	2.3×10^{15}	2.52×10^{-18}	1.53×10^{9}	1.41×10^{9}	-
	11.51	2.3×10^{15}	2.60×10^{-18}	1.53×10^{9}	1.41×10^{9}	-
	11.17	2.3×10^{15}	2.63×10^{-18}	1.53×10^{9}	1.41×10^{9}	-

Supplementary References

1. Nie, W. *et al.* High-efficiency solution-processed perovskite solar cells with millimeter-scale grains. *Science (80-.).* 347, 522 LP – 525 (2015).

2. De, A. & Rao, K. V. Dielectric properties of synthetic quartz crystals. *J. Mater. Sci.* 23, 661–664 (1988).

3. Moses, D., Wang, J., Yu, G. & Heeger, A. J. Temperature-independent photoconductivity in thin films of semiconducting polymers: Photocarrier sweep-out prior to deep trapping. *Phys. Rev. Lett.* 80, 2685 (1998).

4. Moses, D., Soci, C., Chi, X. & Ramirez, A. P. Mechanism of carrier photogeneration and carrier transport in molecular crystal tetracene. *Phys. Rev. Lett.* 97, 67401 (2006).

5. Lee, C. H., Yu, G., Moses, D. & Heeger, A. J. Picosecond transient photoconductivity in poly (p-phenylenevinylene). *Phys. Rev. B* 49, 2396 (1994).

6. Ganichev, S. D. *et al.* Distinction between the Poole-Frenkel and tunneling models of electric-field-stimulated carrier emission from deep levels in semiconductors. *Phys. Rev. B* 61, 10361 (2000).

7. Giorgi, G., Fujisawa, J.-I., Segawa, H. & Yamashita, K. Small Photocarrier Effective Masses Featuring Ambipolar Transport in Methylammonium Lead Iodide Perovskite: A Density Functional Analysis. *J. Phys. Chem. Lett.* 4, 4213–4216 (2013).
